# Extracellular vesicles derived from *Trichinella Spiralis* larvae promote the polarization of macrophages to M2b type and inhibit the activation of fibroblasts

**DOI:** 10.3389/fimmu.2022.974332

**Published:** 2022-09-21

**Authors:** Ji Wu, Yao Liao, Dinghao Li, Zifeng Zhu, Lichao Zhang, Zhongdao Wu, Ping He, Lifu Wang

**Affiliations:** ^1^ Medical Department of Xizang Minzu University, Xianyang, China; ^2^ Department of Parasitology, Zhongshan School of Medicine, Sun Yat-sen University, Guangzhou, China; ^3^ Key Laboratory of Tropical Disease Control, Ministry of Education, Sun Yat-sen University, Guangzhou, China; ^4^ Provincial Engineering Technology Research Center for Biological Vector Control, Guangzhou, China; ^5^ Guangzhou Key Laboratory for Clinical Rapid Diagnosis and Early Warning of Infectious Diseases, KingMed School of Laboratory Medicine, Guangzhou Medical University, Guangzhou, China

**Keywords:** *Trichinella spiralis*, extracellular vesicles, microRNA, macrophages, fibroblasts

## Abstract

*Trichinella spiralis* (*T. spiralis*) is a globally distributed food-borne parasite that can coexist with the host for a long time after infection. *Trichinella*-derived secretions can regulate the immune response and fibroblasts of the host, but the specific mechanisms involved are still unclear. The purpose of this study was to investigate the role of *T. spiralis* larvae-derived extracellular vesicles (EVs) and their key miRNAs in the process of *T. spiralis*–host interaction. In this study, we found that the EVs of *T. spiralis* larvae, as well as miR-1-3p and let-7-5p, expressed in *T. spiralis* larvae-derived EVs, can promote the polarization of bone marrow macrophages to M2b type while inhibiting the activation of fibroblasts. These findings will contribute to further understanding of the molecular mechanisms underlying *T. spiralis*–host interactions.

## Introduction

*Trichinella spiralis* is a globally distributed zoonotic parasite that can cause trichinosis (1–3). It is widely distributed worldwide, including Europe, South America, North America, and Southeast Asia (3, 4). The global prevalence of the disease is difficult to assess (5), but more than 40 million people in China are still at risk of contracting *Trichinella* (6). *Trichinella* were reported to infect a broad range of mammals (3, 6, 7). *Trichinella* larvae parasitize in the host skeletal muscle cells, and *Trichinella* larvae encyst in a collagen capsule, muscle cells proliferate as they are transformed into’nurse cells’ for the larvae (8). *T. spiralis* can be encapsulated in the skeletal muscles of the host, and alternative activated macrophages (M2) play a critical role in *T. spiralis* infection (9). To avoid the immune response of the host, the *T. spiralis* larvae secrete anti-inflammatory substances, such as nuclear factor erythroid 2-related factor-2 (10), *T. spiralis* thioredoxin peroxidase-2 (11), and *T. spiralis* extracellular vesicles (12), to reduce the inflammatory response of the host. To coexist with the host for a long time, the secreted products of *T. spiralis* larvae may have the effect of inhibiting the activation of host fibroblasts. However, in the process of *Trichinella*–host interaction, the key molecules and detailed mechanisms of the anti-inflammatory and inhibiting the activation of fibroblasts of *Trichinella* larvae are still unclear.

Extracellular vesicles (EVs) are vesicles secreted by living cells, containing nucleic acids, proteins, lipids, and other active substances (13–15). EVs are involved in information exchange and regulation between cells (14), and some EVs, including parasite EVs, can even achieve cross-species regulation (16–18). Few studies have shown that EVs secreted by *T. spiralis* can regulate the host immune response (19, 20) and affect macrophage polarization (12), but the specific mechanisms have not been elucidated.

MicroRNA is a type of small RNA that can efficiently regulate gene expression (21, 22), and it plays a key role in the host–parasite interaction (23). Studies have shown that miRNA does not only participate in the communication between cells in the body (24, 25), but can also be transferred to other species through EVs to achieve cross-species regulation (26). For example, miR-let-7-5p derived from *Cysticercus cellulosae* EVs promotes M2 polarization of macrophages by inhibiting the expression of c/ebp δ2 (27); *Tso*-let-7-5p secreted by *Taenia solium* can significantly reduce the levels of IL-6, TNF, IL-12, and other inflammatory factors of macrophages (28); *Emu*-let-7-5p secreted by *Echinococcus multilocularis* significantly reduces the level of IL-1α in macrophages, and at the same time decreases the levels of RIPK1 and NF-kB, which are key components of the LPS/TLR4 signaling pathway in cells (29); *Leishmania donovani* changes the immune response of host macrophages through a complex miRNA mechanism, thereby promoting parasite anti-inflammatory response, which is vital to survival (30). *Schistosoma japonicum* eggs can inhibit the liver fibrosis of the host through high levels of *sja*-mir-71a (23). To make cross-species regulation more smoothly, many miRNAs secreted by parasites are consistent with the host miRNA sequence (27), which may be the result of long-term coevolution between the parasite and the host. However, few studies have analyzed the role of *T. spiralis* miRNA in host immunity and fibroblasts.

Therefore, this study focuses on the specific effects of EVs derived from *T. spiralis* (Ts-EVs) and their key miRNAs on the host immune response and host fibroblasts to further understand the molecular mechanisms underlying *T. spiralis*–host interactions.

## Materials and methods

### Animals and ethics

BALB/c mice (Male, 6 weeks, 18–20 g) and KM mice (Male, 6 weeks, 20–25 g), were purchased from Guangdong Experimental Animal Center. All animal experimental procedures were approved by the Animal Research Ethics Committee of Sun Yat-Sen University and comply with the “Guidelines for the Care and Use of Laboratory Animals” of the National Institute of Health of China.

### Extraction and purification of extracellular vesicles of *T. spiralis* larvae

Each mouse was infected with 350 *T. spiralis* larvae. The mice were sacrificed 35 days postinfection and their whole muscles were put into an artificial digestion solution (1% pepsin + 1% concentrated hydrochloric acid) for 3–4 h (37°C constant temperature shaker). The pure *T. spiralis* larvae were collected using the natural sedimentation method, and washed repeatedly with PBS (containing 100 U/mL penicillin and 100 ug/mL streptomycin) until clear. The larvae of *T. spiralis* were cultured in RPMI 1640 medium (containing 0.5% EV-free serum) for 72 h, and the culture supernatant was collected every 24 h. The culture supernatant was centrifuged at a low speed (700 × g for 30 min at 4°C) to remove cell debris (15 mL polypropylene tube, swinging bucket rotor, model A-4-44, 5804R refrigerated centrifuge, Eppendorf, Germany), and the resulting supernatant was centrifuged at 3,500 × g for 30 min at 4°C (15 mL polypropylene tube, swinging bucket rotor, model A-4-44, 5804R refrigerated centrifuge, Eppendorf, Germany). The supernatant was transferred into a Quick-Seal centrifuge tube (Beckman Coulter, USA) and centrifuged at 100,000 × g for 2 h at 4°C in an Optima L-100xp tabletop ultracentrifuge (swinging bucket rotor, model SW40 Ti, Optima L-100xp, Beckman Coulter, USA).

After resuspension in PBS, it was centrifuged at 100,000 × g and 4°C for 2 h to wash the Ts-EVs (swinging bucket rotor, model SW40 Ti, Optima L-100xp, Beckman Coulter, USA), which were then stored in a refrigerator at –80°C for later use (31, 32).

### Extraction of *T. spiralis* soluble antigens

The purified *T. spiralis* larvae were concentrated and transferred to 1.5 mL tubes (Eppendorf, Germany), and resuspended in PBS. An equal number of magnetic beads was added to the tube, and the *T. spiralis* larvae were homogenized for 5 min at 4°C. Subsequently, the homogenate was centrifuged at 12,000 × g for 20 min (at 4°C), and the supernatant was recovered and distributed into multiple EP tubes.

### Transmission electron microscopy, and nanometer particle size analysis

The Ts-EVs were identified using negative-staining transmission electron microscopy (TEM) and nanoparticle tracking analysis (NTA). The EVs were adsorbed onto the copper grid and then immersed in 2% glutaraldehyde. Subsequently, the copper mesh was negatively dyed with a 3% (w/v) phosphotungstic acid aqueous solution for 1 min. The copper mesh was observed using a TEM (FEI Tecnai G2 Spirit Twin) and photographed to record the shape and size of the Ts-EVs. At the same time, NanoSight NS300 (Malvern Instruments, UK) was used to analyze and fit the Ts-EVs several times to observe the overall distribution and peak value of the Ts-EVs.

### Extraction of primary macrophages

The BALB/c mice were sacrificed through cervical dislocation and soaked in 75% alcohol for 5 min. Subsequently, their femurs and tibias were removed, and bone marrow cells were washed with RPMI 1640 basal medium (Gibco, Germany) to make a single cell suspension. The monocytes were then differentiated in macrophages for 6 days in RPMI 1640 medium [containing 10% fetal bovine serum, 100 U/mL penicillin (Sigma, Germany), 100 µg/mL streptomycin (Sigma, Germany), and 20 ng/mL recombinant mouse macrophage-colony stimulating factor (M-CSF) (PeproTech, USA)]. The prepared bone-marrow-derived macrophages (BMDMs) were detached with trypsin (Gibco, Germany) and transferred to new six-well plates (1 × 10^6^ per well). The BMDMs were treated with *T. spiralis* soluble antigens (10 µg/mL), Ts-EVs (10, 20, and 40 µg/mL), IL-4 (20 ng/mL), IL-1β (20 ng/mL), NC mimic, miR-1-3p mimic, and clet-7-5p mimic; after incubation for 24 h, the cells were harvested.

### Cultivation of human kidney fibroblasts (HKF)

Human kidney fibroblasts (HKF) (Otwo Biotech, China) were cultured in DMEM (Gibco, Germany) complete medium (DMEM + 10% FBS + 100 U/mL penicillin and 100 µg/mL streptomycin, 1 × 10^6^ per well). The HKF were treated with TGF-β (10 µg/mL), Ts-EVs (10, 20, and 40 µg/mL), NC mimic, miR-1-3p mimic, and clet-7-5p mimic. After incubation for 24 h, the cells were harvested.

### Immunofluorescence analysis

Muscle tissues of *T. spiralis*-infected mouse leg were fixed in 4% neutral-buffered formalin and embedded in paraffin. The sections were deparaffinized by baking and then dehydrated using xylene and ethanol. After blocking with bovine serum albumin (BSA), the sections were incubated with F4/80 (1:100, Abcam Cambridge, UK) and α-SMA (1:100, Abcam Cambridge, UK) antibodies overnight at 4°C. The sections were then incubated with the indicated Alexa Fluor-conjugated secondary antibodies. API was used to detect nuclei. Between all steps, samples were washed three times using PBS for 5 min each. The sections were visualized using LSM 800 laser scanning confocal microscope (Zeiss, Germany).

### RNA extraction and real-time quantitative PCR (qRT-PCR)

Total RNAs were isolated from the macrophages and HKF using Trizol reagent (Invitrogen, USA). Using the manufacturer’s instructions, the RNA was first reverse transcribed into complementary DNA (cDNA). Subsequently, SYBR Green QPCR Master Mix (TaKaRa, Japan) was used for real-time quantitative PCR. The reaction process comprised initial denaturation at 95°C for 30 s, 95°C for 5 s, 60°C amplification for 30 s, and 45 cycles of amplification. The 2^−ΔΔCT^ method was used to calculate fold change, and GAPDH and β-actin were used as internal controls. IL-1β, IL-6, IL-10, IL-12, IL-23, TNF-α, TGF-β, Arg-1, iNOS, CCL-1, CCL-17, CXCL-13, VEGF, α- SMA, collagen I, collagen III, collagen IV, collagen VI, GAPDH, and β-actin were used. The primers are shown in **Supplementary Table 1**.

### Cellular protein extraction and western blotting

RIPA buffer with proteinase inhibitor was used to lyse the cells. The proteins were analyzed using SDS–PAGE and transferred to PVDF membranes. After the blocking, the membranes were incubated at 4°C overnight with antibodies to Arg-1, iNOS, TGF-β, TNF-α, IL-10, α-SMA, collagen I, collagen III, collagen IV, collagen VI, GAPDH, and β-actin, followed by incubation with a horseradish peroxidase-linked antibody. The immunoreactive bands were visualized using a chemiluminescence detection system (Amersham, USA). The bands intensity was analyzed by Image J.

### sRNA sequencing analysis of Ts-EVs

Total RNA was isolated from the Ts-EVs and subjected to quantitative and qualitative analyses to ensure the use of qualified samples for sequencing and analysis. The total RNA content of the sample was 7.65 ng, which was used as the input material for generating the small RNA library. Following cluster generation, the library preparations were sequenced on an Illumina Hiseq 2500 platform (Illumina, USA) and paired-end reads were generated. The raw reads of the sample reached 18.65 M or more, and the Q30 base percentage was 75.29% or more. After sequencing, the data were subjected to quality control analysis, and clean reads were compared with reference genome sequences (*Caenorhabditis elegans*) using Bowtie (33). Reads from the reference genome were compared with the miRbase and Rfam databases to obtain known miRNA and ncRNA annotation information, respectively (34). The data were subjected to sequence comparison analysis, target gene prediction and function annotation, quantitative analysis of miRNA levels, and GO and KEGG enrichment analysis.

### The synthesis of mimics

RIBOBIO Inc. (China) synthesized the miRNA-1-3p mimic, let-7-5p mimic, and negative control (NC) mimic.

### Statistical analysis

SPSS 16.0 was used to analyze the data. Differences between groups were analyzed using one-way ANOVA, and *P* < 0.05 was considered significant. The GraphPad Prism software was used to process the data into a histogram for display.

## Results

### Isolation and identification of EVs

**Figure 1:**
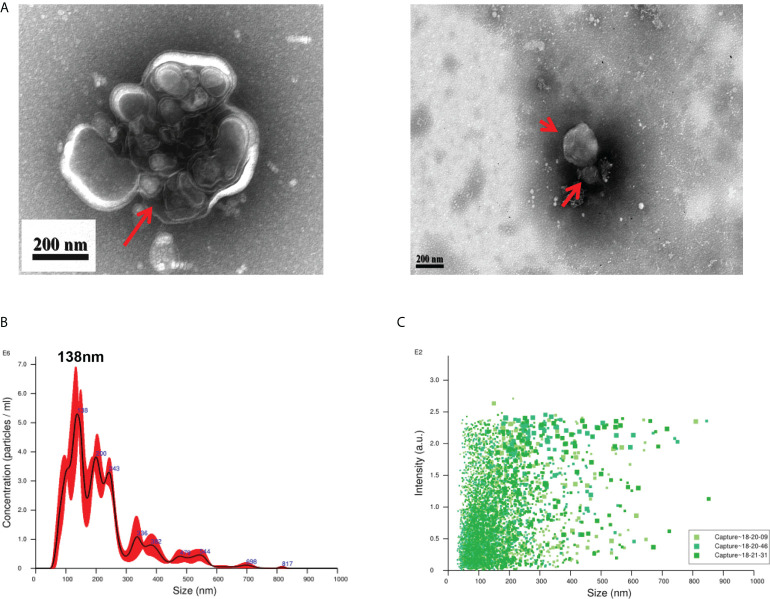
Purification of extracellular vesicles from the culture supernatant of *Trichinella spiralis* larvae. **(A)** Extracellular vesicles of *T. spiralis* (TS-EVs) were analyzed using a negative-staining transmission electron microscope. **(B)** The size distribution and concentration of TS-EVs was analyzed using NanoSight NS300. **(C)** The intensity of TS-EVs was analyzed using NanoSight NS300.

EVs derived from *T. spiralis* larvae were analyzed using negative-staining TEM. Negative-staining TEM revealed that the EVs are round or quasi-circular vesicles with diameters ranging from 30–300 nm (**Figure 1A**). This suggests that we have isolated EVs with typical morphology. The particle size distribution of the EVs was analyzed using NAT. The results showed that the EVs were concentrated in the range of 30–300 nm, and a peak of 138 nm was detected (**Figures 1B, C**). This shows that we successfully extracted and concentrated Ts-EVs.

### EVs regulate macrophage polarization to type 2b

**Figure 2:**
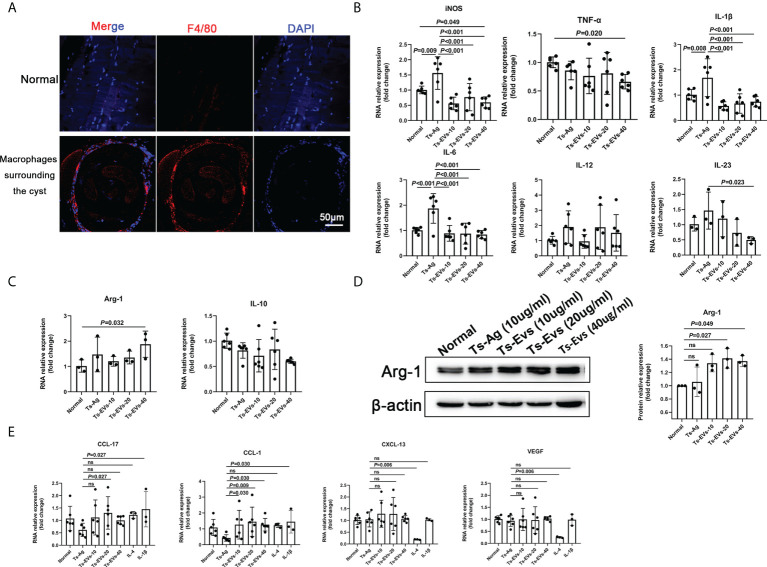
EVs (10, 20, 40 µg/mL) secreted by *T. spiralis* and *T. spiralis* soluble antigens (10 µg/mL) were used to stimulate mouse bone marrow-derived macrophages (BMDMs), and real-time fluorescent quantitative PCR (qRT-PCR) and western blotting were used to detect the levels of related inflammatory cytokines. **(A)** Macrophages surrounding the cyst of larvae were labeled with F4/80 antigen and observed using immunofluorescence. **(B)** The M1-type macrophage marker factors were analyzed using RT-PCR. **(C)** The M2-type macrophage marker factors were analyzed using RT-PCR. **(D)** Arg-1 was analyzed using western blotting. **(E)** The M2-type macrophage subtype marker factors were analyzed using qRT-PCR, IL-4 (M2a), and IL-1β as positive controls (M2b). ns, no significant.

We found that the muscle cells parasitized by *T. spiralis* were accompanied with a large number of macrophage infiltration (**Figure 2A**). To determine this, we used Ts-EVs and *T. spiralis* soluble antigens (Ts-Ag) to stimulate mouse BMDMs cells. We found that compared with the normal group and Ts-Ag group, the levels of iNOS, TNF-α, IL-1β, IL-6, and IL-23 decreased in the Ts-EVs stimulation group (**Figure 2B**). However, the level of Arg-1 increased after treatment with Ts-EVs (**Figures 2C, D**). These results indicate that the EVs of *T. spiralis* larvae can regulate the polarization of macrophages to the M2-type.

Depending on the activating stimulus, M2 macrophages can be classified as M2a, M2b, M2c, or M2d subtypes (35). The different subclasses of M2 macrophages secrete different cytokines, chemokines, and mediators (M2a: CCL-17, M2b: CCL-1, M2c: CXCL-13, and M2d: VEGF) (35). Interestingly, we found that CCL-1 increased significantly after treatment with Ts-EVs (**Figure 2E**). However, there were no significant changes in CCL-17, CXCL-13, and VEGF after treatment with Ts-EVs. These results indicate that EVs cause macrophages to differentiate into the M2b type.

### EVs inhibit HKF activation

**Figure 3:**
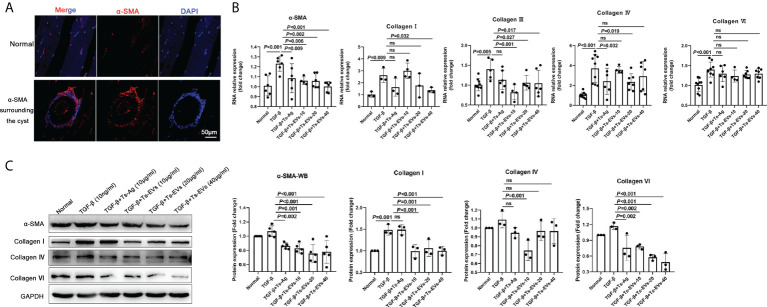
EVs (20 and 40 µg/mL) secreted by *T. spiralis* and *T. spiralis* soluble antigens (10 µg/mL) were used to stimulate human kidney fibroblasts (HKF), and qRT-PCR and western blotting were used to detect the levels of the fibrosis-related factors. **(A)** Fibroblasts surrounding the cyst of the larvae were labeled with α-SMA and observed using immunofluorescence. **(B)** The fibroblast activating factors were analyzed using RT-PCR. **(C)** The fibroblast activating factors were analyzed using western blotting. ns, no significant.

α-SMA is the marker of fibroblast activation. Through immunofluorescence detection, we found a large amount of α-SMA around *T. spiralis* larvae in the late stage of *Trichinella* parasitism (**Figure 3A**). To determine whether the EVs of *T. spiralis* larvae can regulate the host fibroblasts, we used Ts-EVs and Ts-Ag to stimulate HKF. Real-time PCR analysis showed that compared with the normal group, the levels of α-SMA, collagen I, and other fibrotic factors (collagen III, collagen IV, and collagen VI) in the Ts-EVs stimulation group decreased significantly, and the difference was significant (**Figure 3B**). The α-SMA, collagen I, collagen IV, and collagen VI protein levels were confirmed using western blotting, and the results were consistent with those of real-time PCR (**Figure 3C**). This indicates that EVs from *T. spiralis* larvae can inhibit the activation of human HKF, suggesting that Ts-EVs may participate in the host fibrosis reaction process.

### EVs component analysis

**Figure 4:**
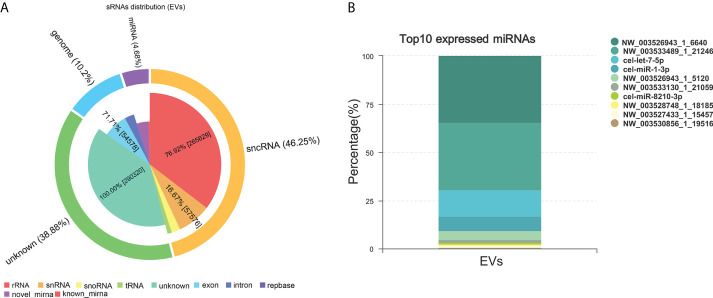
Small RNA sequencing. **(A)** Analysis of small RNA components in Ts-EVs (miRNA:4.68%, sncRNA:46.25, genome: 10.2%, unknown:38.88). **(B)** Top 10 highly expressed miRNAs in *T. spiralis* EVs. Among the known miRNAs of Ts-EVs, miRNA-1-3p and let-7-5p were the most highly expressed.

miRNAs, especially those in EVs, mediate paracrine and endocrine communication between different tissues and thus modulate gene expression and the function of distal cells (36). To identify the components responsible for the regulation of macrophage polarization to type 2 and inhibit HKF activation effects of Ts-EVs, we conducted small RNA sequencing analysis on Ts-EVs and found 64 miRNAs (10 of which are known miRNAs and 54 are unknown miRNAs) (**Figures 4A, B**). Among the known miRNAs of Ts-EVs, miRNA-1-3p and let-7-5p were the most highly expressed (**Table 1**).

**Table 1:** Top 10 highly expressed miRNAs in *T. spiralis* extracellular vesicles.

miRNA	Expression (Read counts)
NW_003533489.1_21246 NW_003526943.1_6640 cel-let-7-5p cel-miR-1-3p NW_003526943.1_5120 NW_003533130.1_21059 cel-miR-8210-3p NW_003528748.1_18185 NW_003527433.1_15457 NW_003530856.1_19516	331678.0229 331678.0229 133849.1743 71139.8545 44693.3826 11086.7306 9238.9421 9123.4554 7968.5876 6755.9764

### MiRNA-1-3p and let-7-5p can regulate the polarization of macrophages to M2b type, and inhibit HKF activation

**Figure 5:**
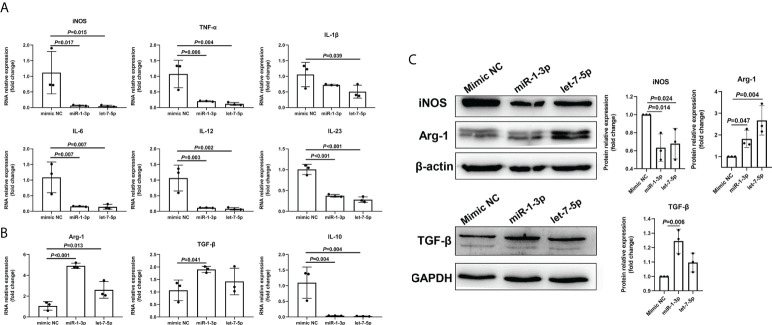
miR-1-3p mimic (50 nM) and let-7-5p mimic (50 nM) were used to stimulate mouse BMDMs, and qRT-PCR and western blotting were used to detect the levels of related inflammatory cytokines. **(A)** The M1-type macrophage marker factors were analyzed using qRT-PCR. **(B)** The M2-type macrophage marker factors were analyzed using qRT-PCR. **(C)** The macrophage marker factors were analyzed using western blotting.

**Figure 6:**
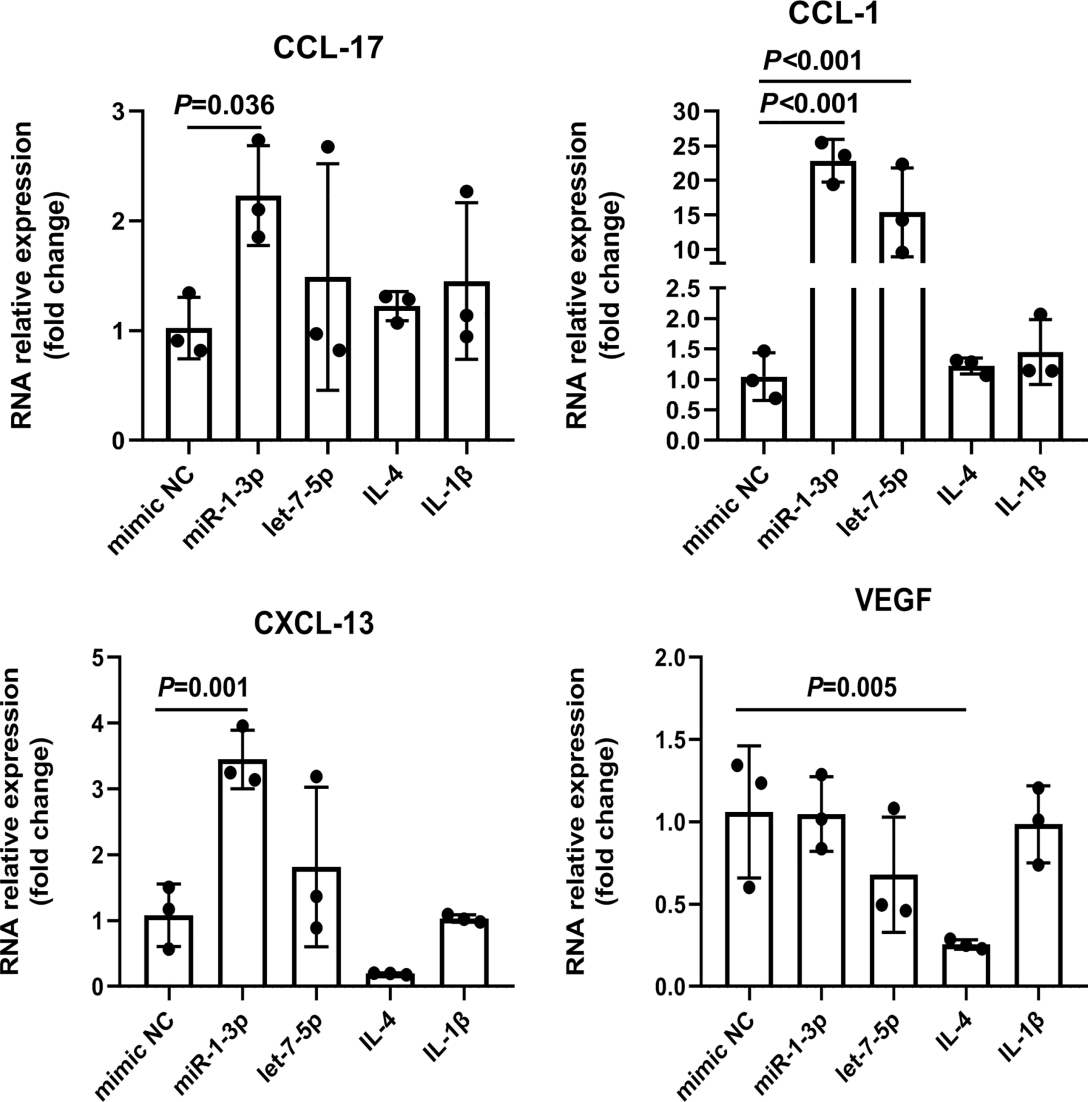
miR-1-3p mimic (50 nM) and let-7-5p mimic (50 nM) were used to stimulate mouse BMDMs, and the M2-type macrophage subtype marker factors were analyzed using qRT-PCR, using IL-4 (M2a) and IL1β (M2b) as positive controls. The housekeeping gene GAPDH was used as an internal control.

To explore the role of Ts-EVs in regulating macrophages and HKF, we synthesized miRNA mimics of miRNA-1-3p and let-7-5p. The results of macrophage studies showed that compared with the NC mimic group, the levels of iNOS, TNF-α, IL-1β, IL-6, IL-12, IL-23, and IL-10 decreased significantly in the miRNA-1-3p and the let-7-5p mimic groups, while the levels of the anti-inflammatory factors Arg-1 and TGF-β increased significantly (**Figures 5A-C**), and the difference was significant (*P* < 0.05). These results show that miRNA-1-3p and let-7-5p can regulate the polarization of macrophages to the M2 type. In addition, the levels of CCL-1 in the miRNA-1-3p and let-7-5p mimic groups also increased (**Figure 6**), suggesting that the polarization direction of macrophages was M2b.

**Figure 7:**
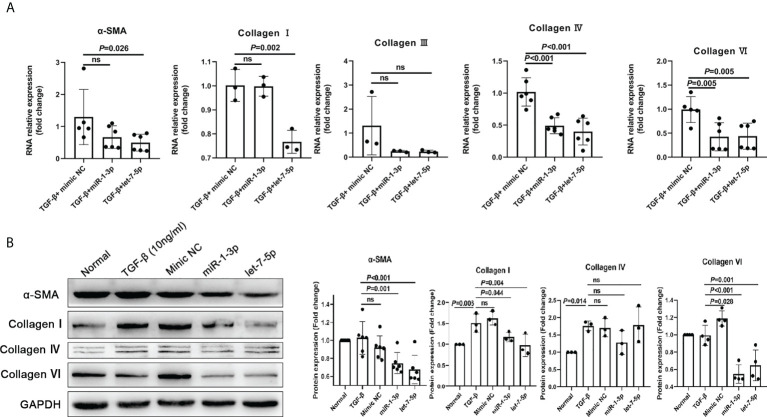
miR-1-3p mimic (50 nM) and let-7-5p mimic (50 nM) were used to stimulate human fibroblasts, and qRT-PCR and western blotting were used to detect the levels of the fibrosis-related factors. **(A)** Results of qRT-PCR. **(B)** Results of western blotting. ns, no significant.

The results of the HKF study showed that compared with the NC mimic group, the levels of α-SMA, collagen I, collagen IV, and collagen VI in the miRNA-1-3p and let-7-5p mimic groups were significantly downregulated (**Figures 7A, B**). This shows that miRNA-1-3p and let-7-5p have a significant inhibitory effect on HKF activation. This suggests that miRNA-1-3p and let-7-5p may be key factors for EVs to inhibit the activation of fibroblasts.

## Discussion

*T. spiralis* is a food-borne parasite can be found worldwide (37, 38). Studies have shown that the excretion/secretion products of *Trichinella* larvae can cause a Th2-type immune response and induce the transformation of macrophages to the M2-type (39, 40). However, the specific mechanisms are not clear. In this study, we found that Ts-EVs can promote macrophages to the M2b type polarization and inhibit the activation of fibroblasts, and these effects are closely related to the high levels of miR-1-3p and let-7-5p in Ts-EVs.

Macrophages are important immune cells and have a direct regulatory effect on the immune response (16). Macrophages can generally be divided into pro-inflammatory macrophages (type M1) and anti-inflammatory macrophages (type M2) (16, 41). When the pathogen *Trichinella* invades the host, it activates the host immune system, and induces a large number of inflammatory cells to infiltrate. To avoid the attack of host immune cells, *T. spiralis* suppresses the host immune response by secreting related substances and induces macrophages to polarize to the M2 type. However, the specific components and their mechanisms are still unclear.

EVs are vesicle-like bodies with biological functions, and are secreted by living cells; they contain various biologically active molecules such as proteins, lipids, carbohydrates, and nucleic acids (13, 16, 42). Studies have shown that when parasites infect the host, they can regulate the host immune microenvironment by secreting EVs to achieve long-term parasitism (16, 43, 44). For example, *Leishmania* EVs can regulate the activity of host NF-κB (45); *Fasciola hepatica* EVs induce DC cell production of a unique phenotype that can inhibit the secretion of IL-2 by T cells (46, 47). There are also studies showing that Ts-EVs can promote the infiltration of M2-type macrophages into the colon (12, 40). It is speculated that Ts-EVs can inhibit the polarization of M1-type macrophages, but there is a lack of systematic cellular experimental support. Here, we showed that Ts-EVs can inhibit the levels of IL-1β, iNOS, TNF-α, IL-6, and IL-23 in macrophages. At the same time, it promoted the levels of anti-inflammatory factors Arg-1 and TGF-β in macrophages. These results suggest that Ts-EVs can stimulate macrophages to polarize to the M2-type. To explore the specific subtypes of macrophage polarization, we detected the chemokines CCL-1, CCL-17, CXCL-13, and VEGF that are highly expressed by the four subtypes (M2a, M2b, M2c, and M2d) of macrophages (35). The results show that after treatment with Ts*-*EVs, macrophages significantly released CCL-1, which suggests that Ts-EVs may induce macrophages to polarize to the M2b type. EVs of helminths are packed with specific cargo with immunomodulatory properties, including proteins, nucleic acids, and lipids (18). EVs are a vehicle for the transport of genetic material for cell-to-cell communication and nucleic acids are a ubiquitous constituent of EV cargo (48). miRNAs, especially those in EVs, mediate paracrine and endocrine communication between different tissues and thus modulate gene expression and the function of distal cells (36). Therefore, we further analyzed the miRNAs in TS-EVs, and found that similar to Ts-EVs, miR-1-3p and let-7-5p induce macrophages to polarize to the M2b type. These results suggest that *T. spiralis* may secrete Ts-EVs with high levels of miRNA miR-1-3p and let-7-5p to regulate the polarization of host macrophages to the M2b type, thereby evading the host’s immune response to achieve the purpose of long-term parasitism. IL-10 is an anti-inflammatory cytokine. However, in this study, we found that miR-1-3p and let-7-5p down-regulated the expression of IL-10, which is an interesting phenomenon and worthy of further study.

Fibroblasts are essential in the formation of *T. spiralis* larvae cysts (49). Studies have shown that when *T. spiralis* larvae infect host muscle cells, the muscle cells and fibroblasts synthesize collagen fibers to wrap the larvae to form a capsule (49). In addition, the collagen capsule is also a component of *T. spiralis* nurse cell, which enable the larva can be ingested by the next potential host. However, the specific mechanisms are rarely reported in the study. Most studies have focused on *T. spiralis* and host immune cells, ignoring the important role of fibroblasts in the parasitic process of *T. spiralis*. We used Ts-EVs and mimic miR-1-3p and let-7-5p to stimulate HKF, and observed the activation of HKF. Following the stimulation of HKF by Ts-EVs and mimic miR-1-3p and let-7-5p, the levels of the fibrotic factors in HKF decreased significantly. Interestingly, Ts-EVs can stimulate macrophages to polarize to the M2-type and highly express TGF-β, and TGF-β activates HKF, prompting them to secrete collagen I and α-SMA (50). However, Ts-EVs inhibited HKF activation and reduced the levels of collagen I and α-SMA. This contradiction may be a balance between the long-term coexistence of the host and the parasite.

Taken together, we speculate that when *T. spiralis* infects the host, *T. spiralis* larvae secrete a large number of EVs containing miR-1-3p and let-7-5p. These EVs induce host macrophages to polarize to the M2-type, thereby weakening the immune response against *T. spiralis*. At the same time, macrophages highly express TGF-β, which triggers the fibrosis of host muscle cells (9), wraps the *T. spiralis* larvae to form collagen capsules, and eventually calcify and die. However, *T. spiralis* larvae produce a large amount of miR-1-3p and let-7-5p to inhibit the fibrotic process of the host, thereby prolonging its existence in the host and increasing the possibility of infecting other hosts.

In summary, our data show that the EVs of *T. spiralis* larvae, as well as miR-1-3p and let-7-5p expressed in *T. spiralis* larvae-derived EVs, can promote the polarization of bone marrow macrophages to the M2 type, while inhibiting the activation of fibroblasts. These findings will contribute to further understanding of the molecular mechanisms underlying *T. spiralis*–host interactions.

## Data availability statement

The data presented in the study are deposited in the GenBank repository, accession number 2621734.

## Ethics statement

The animal study was reviewed and approved by Animal Research Ethics Committee of Sun Yat-Sen University and comply with the “Guidelines for the Care and Use of Laboratory Animals” of the National Institute of Health of China.

## Author contributions

JW, LW, YL, PH, DL, ZZ, and ZW drafted this manuscript. JW and YL performed the experiments, analyses, and interpretation of the data. DL helped the aforementioned authors to develop the experiments. ZZ and LZ provided final approval of the version to be published. All authors discussed the complete dataset to establish an integral and coherent analysis.

## Funding

This work was supported by National Natural Science Foundation of China (Nos. 81902081, 81871682, and 81860365), the Natural Science Foundation of Guangdong Province (Nos. 2020A1515011573, 2019A1515012068, and 2021A1515010976), Guangzhou key laboratory for clinical rapid diagnosis and early warning of infectious diseases (202102100003).

## Conflict of interest

The authors declare that the research was conducted in the absence of any commercial or financial relationships that could be construed as a potential conflict of interest.

## Publisher’s note

All claims expressed in this article are solely those of the authors and do not necessarily represent those of their affiliated organizations, or those of the publisher, the editors and the reviewers. Any product that may be evaluated in this article, or claim that may be made by its manufacturer, is not guaranteed or endorsed by the publisher.
