# Supplementary material for: Extracellular vesicles derived from Trichinella Spiralis larvae promote the polarization of macrophages to M2b type and inhibit the activation of fibroblasts
Source: Front Immunol. 2022 Sep 21;13:974332. doi: 10.3389/fimmu.2022.974332 (PMC9532625; doi:10.3389/fimmu.2022.974332)
Supplement: Supplementary file 1 [file Table_1.docx]

**Supplementary table 1:** Primers used for real-time PCR analysis.

| Genes | Primer | Sequence(5′-3′) |
| --- | --- | --- |
| α-SMA (human) | Forward primer | CCAGGGCTGTTTTCCCATCC |
|  | Reverse primer | GCTCTGTGCTTCGTCACCCA |
| Collagen Ⅰ(human) | Forward primer | GATTCCCTGGACCTAAAGGTGC |
|  | Reverse primer | AGCCTCTCCATCTTTGCCAGCA |
| Collagen Ⅲ (human) | Forward primer | TGGTCTGCAAGGAATGCCTGGA |
|  | Reverse primer | TCTTTCCCTGGGACACCATCAG |
| Collagen Ⅳ (human) | Forward primer | AGGATTTACCGGACCACCAG |
|  | Reverse primer | TGGTCACCCTTGTCACCTTT |
| Collagen Ⅵ (human) | Forward primer | GCCTTCCTGAAGAATGTCACCG |
|  | Reverse primer | TCCAGCAGGATGGTGATGTCAG |
| GAPDH (human) | Forward primer | GTCTCCTCTGACTTCAACAGCG |
|  | Reverse primer | ACCACCCTGTTGCTGTAGCCAA |
| CTGF (Mouse) | Forward primer | TGCGAAGCTGACCTGGAGGAAA |
|  | Reverse primer | CCGCAGAACTTAGCCCTGTATG |
| IL-1β (Mouse) | Forward primer | TGGACCTTCCAGGATGAGGACA |
|  | Reverse primer | GTTCATCTCGGAGCCTGTAGTG |
| IL-6 (Mouse) | Forward primer | TACCACTTCACAAGTCGGAGGC |
|  | Reverse primer | CTGCAAGTGCATCATCGTTGTTC |
| IL-10 (Mouse) | Forward primer | CGGGAAGACAATAACTGCACCC |
|  | Reverse primer | CGGTTAGCAGTATGTTGTCCAGC |
| IL-12a (Mouse) | Forward primer | ACGAGAGTTGCCTGGCTACTAG |
|  | Reverse primer | CCTCATAGATGCTACCAAGGCAC |
| IL-23a (Mouse) | Forward primer | CAGCAGCTCTCTCGGAATCTC |
|  | Reverse primer | TGGATACGGGGCACATTATTTTT |
| TNF-α (Mouse) | Forward primer | GGTGCCTATGTCTCAGCCTCTT |
|  | Reverse primer | GCCATAGAACTGATGAGAGGGAG |
| TGF-β (Mouse) | Forward primer | TGATACGCCTGAGTGGCTGTCT |
|  | Reverse primer | CACAAGAGCAGTGAGCGCTGAA |
| Arg-1 (Mouse) | Forward primer | TGTCCCTAATGACAGCTCCTT |
|  | Reverse primer | GCATCCACCCAAATGACACAT |
| iNOS (Mouse) | Forward primer | GAGACAGGGAAGTCTGAAGCAC |
|  | Reverse primer | CCAGCAGTAGTTGCTCCTCTTC |
| CCL-1 (Mouse) | Forward primer | GCTTACGGTCTCCAATAGCTGC |
|  | Reverse primer | GCTTTCTCTACCTTTGTTCAGCC |
| CCL-17 (Mouse) | Forward primer | CGAGAGTGCTGCCTGGATTACT |
|  | Reverse primer | GGTCTGCACAGATGAGCTTGCC |
| CXCL13 (Mouse) | Forward primer | CATAGATCGGATTCAAGTTACGCC |
|  | Reverse primer | GTAACCATTTGGCACGAGGATTC |
| VEGF (Mouse) | Forward primer | CTGCTGTAACGATGAAGCCCTG |
|  | Reverse primer | GCTGTAGGAAGCTCATCTCTCC |
| CTGF (Mouse) | Forward primer | TGCGAAGCTGACCTGGAGGAAA |
|  | Reverse primer | CCGCAGAACTTAGCCCTGTATG |
| GAPDH (Mouse) | Forward primer | CATCACTGCCACCCAGAAGACTG |
|  | Reverse primer | ATGCCAGTGAGCTTCCCGTTCAG |

**Supplementary table 2:** Antibodies List (Western blotting)

| Primary antibody | Working conditions | Catalogue No |
| --- | --- | --- |
| α-SMA | 1:2000 | Proteintech 14395-1-AP (Wuhan, china) |
| Collagen Ⅰ | 1:2000 | Proteintech 14695-1-AP (Wuhan, china) |
| Collagen Ⅲ | 1:1000 | Zenbio R23957 (Chengdu，china) |
| Collagen Ⅳ | 1:1000 | Zenbio 252404 (Chengdu，china) |
| Collagen Ⅵ | 1:1000 | Zenbio 383959 (Chengdu，china) |
| Arg-1 | 1:5000 | Proteintech 16001-1-AP (Wuhan, china) |
| iNOS | 1:1000 | Abcam ab49999 (Cambridge, UK) |
| TNF-α | 1:1000 | Abcam ab1793 (Cambridge, UK) |
| TGF-β1 | 1:1000 | Proteintech 21898-1-AP (Wuhan, china) |
| IL-10 | 1:1000 | Proteintech 20850-1-AP (Wuhan, china) |
| β-actin | 1:5000 | SAB 21338 (MD, USA) |
| GAPDH | 1:5000 | Proteintech 60004-1-Ig (Wuhan, china) |
